# Towards safe and sustainable innovation in nanotechnology: State-of-play for smart nanomaterials

**DOI:** 10.1016/j.impact.2021.100297

**Published:** 2021-01

**Authors:** Stefania Gottardo, Agnieszka Mech, Jana Drbohlavová, Aleksandra Małyska, Søren Bøwadt, Juan Riego Sintes, Hubert Rauscher

**Affiliations:** a European Commission, Joint Research Centre (JRC), Ispra, Italy; b European Commission, DG Research and Innovation, Brussels, Belgium

**Keywords:** Smart nanomaterials, Stimuli-responsive, Nanotechnology, Safe innovation, Safe and sustainable by design, Regulatory preparedness, Regulation of nanomaterials, Circular economy

## Abstract

The European Green Deal, the European Commission's new Action Plan for a Circular Economy, the new European Industrial Strategy and the Chemicals Strategy for Sustainability launched in October 2020 are ambitious plans to achieve a sustainable, fair and inclusive European Union's economy. In line with the United Nations Sustainable Development Goals 2030, these policies require that any new material or product should be not only functional and cost-effective but also safe and sustainable to ensure compliance with regulation and acceptance by consumers. Nanotechnology is one of the technologies that could enable such a green growth. This paper focuses on advanced nanomaterials that actively respond to external stimuli, also known as ‘smart nanomaterials’, and which are already on the market or in the research and development phase for non-medical applications such as in agriculture, food, food packaging and cosmetics. A review shows that smart nanomaterials and enabled products may present new challenges for safety and sustainability assessment due to their complexity and dynamic behaviour. Moreover, existing regulatory frameworks, in particular in the European Union, are probably not fully prepared to address them. What is missing today is a systematic and comprehensive approach that allows for considering sustainability aspects hand in hand with safety considerations very early on at the material design stage. We call on innovators, scientists and authorities to further develop and promote the ‘Safe- and Sustainable-by-Design’ concept in nanotechnology and propose some initiatives to go into this direction.

## Highlights

• Novel, stimuli-responsive (“smart”) nanomaterials are used in food, food packaging, cosmetics and agriculture.
• Smart nanomaterials pose new challenges to safety and sustainability assessment due to their complexity and dynamic behaviour.
• Existing regulatory frameworks are probably not fully prepared to address such challenges.
• Smart nanomaterials should comply with the aims of the European Green Deal and the Chemicals Strategy for Sustainability.
• ‘Safe- and Sustainable-by-Design’ integrates safety, circularity and functionality of materials throughout their life cycle.

## 1 Introduction

The European Green Deal is an ambitious roadmap to a sustainable European Union's economy through a fair and inclusive transition process for all citizens (European Commission, 2019a). It aims at moving to a clean and climate-neutral economy by means of green investments in environmentally friendly technologies and through actions dedicated to biodiversity restoration and pollution reduction. This policy shift has been recently initiated by the European Commission's Circular Economy Action Plan (European Commission, 2020a). This plan intends to establish a competitive and resource and energy efficient economy able to minimise waste and greenhouse gas emissions and to maintain materials, products and resources in a closed loop as long as possible over time (Stahel, 2016), so that the products of today are also the raw materials of tomorrow. A circular economy applies the concept of ‘sustainable development’ (United Nations, 1987) to the entire life cycle and value chain of materials and products enabled by them. The term was coined by the United Nation (Brundtland Commission) in 1987 and defined it as an economic prosperity that *“meets the needs of the present without compromising the ability of future generations to meet their own needs”* (United Nations, 1987), thus promoting a legacy of economic viability, social equity and environmental responsibility for current and future generations. Accordingly, sustainability of a country, community or organisation is commonly conceived as encompassing three interlinked pillars: environmental, economic and social (Purvis, 2019). The same applies when characterising the sustainability of a consumer product or material: each stage of the life cycle of a product or material (raw materials extraction, materials processing, product manufacture, product use, end-of-life disposal) should be optimised to minimise the impacts within the three pillars and to reach a balance reconciling all aspects in a holistic approach (Eason et al., 2011).

This goes into the same direction as the ‘green growth’ and ‘green innovation and technology’ recently advocated by the Organisation for Economic Co-operation and Development (OECD) (Organisation for Economic Co-operation and Development (OECD), 2012; Organisation for Economic Co-operation and Development (OECD), 2013). In particular, the Circular Economy Action Plan is committed to implement the United Nations' Sustainable Development Goals (United Nations, 2015) in all European Union policies and encourages all Member States to do the same at national level. The Green Deal is also a driving force for the European Commission's new Chemicals Strategy for Sustainability (European Parliament, 2020; European Commission, 2020b; European Council, 2019), aimed at promoting responsible innovation in the chemical sector through the development of safer and more sustainable alternatives, and the New Industrial Strategy for Europe (European Commission, 2020c), which highlights the leading role of industry in the transition towards climate neutrality.

**Fig. 1:**
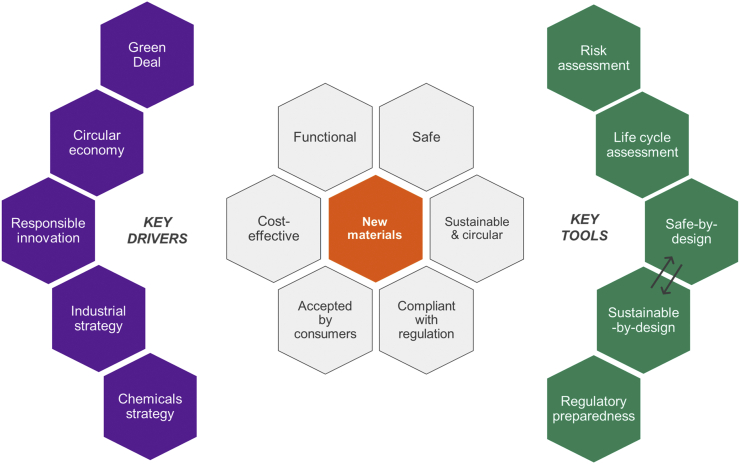
Key drivers and tools for new materials. The European Union's policy initiatives (purple hexagons) driving research, development and production of new materials and key tools (green hexagons) needed to ensure their safety and sustainability. Sustainable-by-design aims to ensure incorporating sustainability aspects very early on at the material design stage hand in hand with safety consideration. The Safe Innovation Approach combines Safe-by-Design with Regulatory Preparedness (Soeteman-Hernandez et al., 2019). (For interpretation of the references to colour in this figure legend, the reader is referred to the web version of this article.)

These policy initiatives aim to address safety and sustainability of a material or product at the early stage of the design process rather than relying on control and measures to retroactively mitigate their impact on human health and the environment. Safety is usually a legal requirement for companies to fulfil to obtain access to the market for their substances and products; for instance, under the European Regulation concerning Registration, Evaluation, Authorisation and Restriction of Chemicals (REACH) (The European Parliament and the Council of the European Union, 2006), a chemical safety assessment is required to ensure that the manufacture and intended uses of a substance do not pose unacceptable risks to human health (workers and consumers) and the environment. The move towards a circular economy, despite several implementation challenges (Olsen, 2019), should be perceived as an important catalyst for research and innovation. Indeed, it stimulates manufacturers to design new materials and products (and associated production processes) in a way that maximise their lifetime and potential for remanufacturing, reuse and recycling, thus reducing the demand for finite resources, waste generation and greenhouse gas emissions (Clark et al., 2016; Keijer et al., 2019) Accordingly, any new material or product should not only be functional and cost-efficient but also safe and sustainable, so as to ensure compliance with regulations, acceptance by consumers and users and, consequently, a fast and successful access to the market (Fig. 1). To this end, the experience that has been gained from decades of work on ‘green chemistry’ (e.g., Anastas and Warner, 1998, Anastas and Warner, 2021; Anastas and Eghbali, 2010) is crucial for enabling a circular economy in this specific sector (Clark et al., 2016; Keijer et al., 2019).

Nanotechnology is often mentioned among those technologies that could enable a green growth (Organisation for Economic Co-operation and Development (OECD), 2013). The term ‘green nanotechnology’, sometimes referred to as ‘green nanomaterials’ or ‘green nanoscience’, concerns the application of the green chemistry principles to the design, manufacturing, use and end-of-life of nanomaterials and nano-enabled products (Dahl et al., 2007; Eckelman et al., 2008; Varma, 2012; Gilbertson et al., 2015; Lu and Ozcan, 2015). Progress has been made in this field, for instance by manufacturing and using less hazardous nanomaterials (e.g. nano-cellulose instead of synthetic nano-polymers) or carrying out synthesis processes that make use of safer and renewable starting materials, reagents and solvents (e.g., Dahl et al., 2007; Lu and Ozcan, 2015). Nevertheless, despite the fact that the scientific community has made significant advancements in the last 15 years in understanding the environmental and toxicological behaviour of nanomaterials, some questions concerning the safety and sustainability of nanotechnology applications in consumer and industrial products remain open, and they become even more challenging when addressing new generations of nanomaterials (Jantunen et al., 2018a; Friedersdorf et al., 2019; Grieger et al., 2019). Recently, regulatory authorities and scientists (e.g., International Risk Governance Council (IRGC), 2007) have signalled possible concerns regarding risk assessment and governance of those advanced nanomaterials that actively respond to external stimuli, also known as ‘smart nanomaterials’. Both smart nanomaterials and smart materials share concerns associated to their functionality. In addition to that, smart nanomaterials bring along all issues and challenges that have been identified for materials at the nanoscale. These materials are already used in products with biomedical, cosmetic and electronic applications, and may soon be introduced in other sectors as well, for instance in agriculture. It seems therefore necessary to promote the development of new smart nanomaterials that are safe and sustainable. This has to be ensured by a robust but flexible regulatory framework that is able to keep pace with innovation without creating unnecessary barriers for industry. The Safe Innovation Approach (SIA) for nanomaterials, combining Safe-by-Design (SbD) in industrial settings and Regulatory Preparedness (RP) by authorities (Soeteman-Hernandez et al., 2019), is a possible way forward to achieve this (Fig. 1). Safety of a nanomaterial in the context of SbD means that (a) the materials are non-hazardous for humans and the environment, (b) the production should aim to eliminate risks at the workplace and to eliminate waste and (c) during the entire life cycle exposure during use of the product should be avoided and efficient recycling and disposal routes should be available (Soeteman-Hernandez et al., 2019; Gottardo et al., 2017). Including sustainability criteria in the SIA can improve societal trust in the industrial innovation process by creating a more inclusive system that involves a broader range of stakeholders in the process and allows for rapid iteration to meet the needs of all actors, including society (Fig. 1).

This review focuses on smart nanomaterials for non-medical applications, both as individual materials and when they are embedded in products. We discuss smart nanomaterials, which are either already on the market (mainly in cosmetics, food, food packaging) or at research and development stages for application in sectors where their use currently is less common or expected in the near future (e.g. in agriculture). The available knowledge and possible concerns regarding their safety, sustainability and related legislative aspects are reviewed and discussed. The paper concludes reporting some open challenges that need to be collectively addressed by scientists, industrial stakeholders and regulators to ensure that the upcoming smart nanomaterials and associated enabled products are safe and sustainable and thus facilitate the realisation of their full socioeconomic potential in line with the European Green Deal in a circular economy.

## 2 What are smart nanomaterials?

The term ‘smart material’ (alternatively, ‘active’, ‘adaptive’ or ‘stimuli-responsive’ material) is used in the scientific literature to refer to a material that timely and reversibly changes certain critical properties during use and activates specific functions upon exposure to one or more external stimuli (Cao et al., 1999; Akhras, 2000; Yoshida and Lahann, 2008). The external stimulus may originate from different sources, for instance changes in the environmental conditions, such as temperature, pH, and light, the application of mechanical forces, electric and magnetic fields, or the presence of and interaction with a specific molecule such as an enzyme or a solvent. Examples include sensors (Setka et al., 2019), actuators and targeted delivery systems (or carriers). Some of these stimuli are naturally occurring in vivo, for example a different pH in different cellular compartments or in some tumour tissues compared to healthy ones. Smart materials can be considered as part of the broader category of ‘advanced materials’ (Umwelt Bundesamt (UBA), 2020). This term currently lacks of a consensus definition in the scientific community but is generally used to cover all materials exhibiting *“novel or enhanced properties that improve performance over conventional products and processes”* (Kennedy et al., 2019), including materials with increased temperature resistance, higher strength and lower weight, or super-plasticity. These novel or enhanced properties are usually obtained by combining several materials (conjugation, hybridisation) or adding specific structures or coating layers to the surface of a material (modification, functionalisation), thus resulting in complex, multi-component hierarchical systems performing a variety of functions. Their properties therefore often result from a combination of the chemical nature of the materials and the intentional structural design. Often, advanced materials are at the nanoscale or have one or more nanoscale entities as components of their structure, for example ‘nanohybrids’ (Aich et al., 2014). In the case of nanostructured advanced materials, the assembly method is also reported to play a role in determining their unique properties (Jortner and Rao, 2002). In particular, smart nanomaterials not only present more complex structures than conventional ones but are designed to have higher dynamism by actively transforming in response to external stimuli, as described for example in example in Plazas-Tuttle et al. (2015). Due to these changing properties, crucial challenges are assessing and predicting the behaviour of smart nanomaterials and their possible toxicological effects after release into the environment and during their whole life cycle.

The International Standardization Organisation (ISO) defines a nanomaterial as a *“material with any external dimension in the nanoscale or having internal structure or surface structure in the nanoscale”*, with nanoscale being the *“length range approximately from 1 nm to 100 nm”* (ISO/TS 20477:2017(en) Nanotechnologies, 2017). The European Commission published a Recommendation on the definition of the term nanomaterial (European Commission, 2011), and this definition or core parts of it are now integrated and binding in several European legal acts, for example in the REACH Regulation (The European Parliament and the Council of the European Union, 2006). There are other legal acts in Europe (The European Parliament and the Council of the European Union, 2009; The European Parliament and the Council of the European Union, 2015) and beyond (U.S. Department of Health and Human Services, Food and Drug Administration, Office of the Commissioner, 2014; Health Canada, 2020), which use their own definitions of nanomaterial or criteria for products of nanotechnology. Hence, if smart nanomaterials fall under the scope of a specific regulatory provision, the respective terminology is relevant, whereas in a general context the European Commission's definition of nanomaterial would apply.

In the categorisation system for nanotechnology products envisaged by Roco (2004), a shift from the development of ‘passive’ nanostructures (first generation, e.g. dispersions of nanoparticles, nanostructured coatings, nanostructured metals, polymers and ceramics) to ‘active’ ones (second generation, i.e. nanostructures changing composition and/or behaviour during their use in response to a stimulus such as smart nanomaterials) was predicted to take place by 2010. Some authors have later on documented a sharp rise of publications on ‘active’ nanostructures in 2006^48^ and then a steady continuous growth until 2010, confirming the predicted shift in focus from passive to active nanotechnology research (Suominen et al., 2016). Actually, a recent literature search focused on adaptive nanohybrids has revealed a near exponential increase in publication number over the period 2004–2014 (Plazas-Tuttle et al., 2015).

Smart nanomaterials can be categorised in different ways. Subramanian et al. (2010) distinguished five categories of active nanostructures based on their structure or function: (1) Remote-actuated (the active principle is remotely activated or sensed); (2) Environmentally-responsive (sensitive to stimuli such as pH, temperature, light, oxidation–reduction, certain chemicals and others); (3) Miniaturized (a conceptual scaling down of larger devices and technologies to the nanoscale); (4) Hybrid (involving uncommon combinations (biotic–abiotic, organic–inorganic) of materials); and (5) Transforming (structures which change irreversibly during some stage of their use or life). Plazas-Tuttle et al. (2015) presented a classification scheme specific for adaptive nanohybrids based on the key environmental stimuli they respond to: (1) pH-responsive; (2) thermo-responsive; (3) photo-responsive; and (4) multi-stimuli-responsive.

Smart nanomaterials fall under the second and the third generations of nanotechnology applications as recently re-defined by the European Chemicals Agency (ECHA) (2019). ECHA published an inventory of 48 second generation (i.e. materials and structures) and 8 third generation (i.e. systems) stimuli-responsive nano-enabled products. About 70% of them are currently on the market; the rest is expected to be placed onto the market in the next 5–10 years as inferred, for instance, from available information on ongoing clinical trials. More than half of the second-generation nano-enabled products are for medical applications (diagnosis and disease treatment) and about one fourth for electronics applications (mainly for displays). Three products (two already on the market) are used for environmental applications (gas detection and contamination remediation). All third-generation products inventoried by ECHA are not on the market yet and again are intended for medical and electronic applications.

However, the peer-reviewed literature reveals that intensive research is ongoing and new smart nanotechnology applications are being developed in other industrial sectors, such as cosmetics, food, food packaging and agriculture, tested in the laboratory and will eventually be put on the market (Box 1).

Box 1 **Examples of smart nanomaterials' applications.**

Smart nanomaterials are used as nanoencapsulates and nanocarriers in controlled release technology and delivery systems for bioactive substances, especially drugs (Vega-Vásquez et al., 2020). In particular, the current COVID-19 crisis has fuelled the demand for effective diagnostic and treatment tools to help the fight against the pandemic and the stimuli-responsive characteristics of smart nanomaterials are being explored for the design of dedicated controlled drug delivery systems, better antigen presentation and immune modulation (Chauhan et al., 2020; Weiss et al., 2020). Advancement in the area of biodegradable nanoparticles may contribute to the development of novel and effective surface coatings resistant to viral adhesion (Cagno et al., 2018).

In addition to disease prevention and therapeutic potential, smart nanomaterials might play a role in diagnosis. Some strategies are being explored for nano-enabled bio-sensing systems as next generation non-invasive disease diagnostics tools, including the use of smart bio-sensing materials for diagnosis at point-of-care applications (Mujawar et al., 2020).

In the last decade, controlled delivery systems have paved their way from medicinal products to cosmetics (Tripura Sundari and Anushree, 2017; Kaul et al., 2018; Santos et al., 2019) and food, and are being considered for application in agriculture. For instance, recently a new family of stimuli-responsive nanocapsules designed to enclose a cosmetic ingredient able to treat different skin conditions (e.g. contact dermatitis, skin photo-damage) has been developed (Aziz et al., 2019) and commercialised (https://www.emissarycosmetics.com/). The PeptiCaps nanocapsules release the active ingredient in the desired location in response to changes of pH or presence of specific enzymes caused by the skin condition to be treated. Likewise, nanoencapsulates are used in some of the so called ‘deodorants on request’ to release a fragrance or a bacteria growth inhibitor in response to the changed pH, temperature or moisture of the skin (Keegan et al., 2012; Hofmeister et al., 2014).

In the food sector nanoencapsulates have been used for different purposes (Jafari, 2020), for instance to prevent nutrients, antioxidant molecules such as ferulic acid and tocopherol (Oehlke et al., 2017), hydrophobic flavouring agents (Fucinos et al., 2014) or natural antimicrobial ingredients (Piran et al., 2017) from breaking down in the body and to allow a slow release at a target specific location or at the presence of a specific molecule.

Lowry et al. (2019) and Kah et al. (2019) have recently reviewed the potential for developing smart nanostructures enabling a targeted (in terms of time, location, dose and form) release and delivery of water, nutrients, agrochemicals and antimicrobials to crops, in order to improve the use efficiency as well as to preserve soil integrity and function. Nanotechnology can also be used in agriculture to develop smart plant sensors for real-time detection and monitoring of plant pathogens and stress conditions (Giraldo et al., 2019).

Stimuli responsive food packaging reacts to stimuli in the food or the environment to enable real time food quality and food safety monitoring or remediation. The most popular use of stimuli responsive nanomaterials in food packaging is the monitoring of gaseous compounds like O_2_, CO_2_ or ethylene present in the headspace of the package (Brockgreitens and Abbas, 2016). Stimuli responsive nanomaterials are also proposed to be used in corrective responsive food packaging due to their changes in such a shrinking, swelling or self-assembly, which can trigger the release of a compound to prevent e.g. microbial growth, off flavours, off odours development, colour changes or nutritional losses (Ghosh et al., 2019).

Brockgreitens and Abbas (2016) envisaged that the responsive packaging will have a tremendous impact on food industry by reducing spoilage, food waste, food recalls, or foodborne illness outbreaks thus contributing to sustainable development and safety.

Alt-text: Box 1

## 3 Are smart nanomaterials sustainable?

Some methodologies for characterising sustainability already exist for nanomaterials and nano-enabled products. For example, LICARA NanoSCAN (Van Harmelen et al., 2016) is a screening tool aimed to assist small and medium enterprises in evaluating environmental, economic and social benefits against risks of nano-enabled products compared to conventional ones with similar function. To this end, Risk Assessment (RA) and Life Cycle Assessment (LCA) are used and resulting indicators visualized on a bar chart for comparison (Van Harmelen et al., 2016). LICARA NanoSCAN is integrated in a wider Sustainable Nanotechnology Decision Support System (SUNDS; https://sunds.gd/), which uses LCA, RA, Economic Assessment and Social Impact Assessment to estimate a set of weighed environmental, social and economic indicators and derive a sustainability score for each alternative (conventional or advanced) material/product (Subramanian et al., 2016). LCA is a well-established and systematic method for assessing the potential environmental impacts of products across their entire life cycles (ISO 14040:2006, 2006). However, LCA case studies on nanotechnology products are currently hindered by knowledge gaps (Gottardo et al., 2017), especially regarding their release into the environment and exposure (Salieri et al., 2018), and remain focused on a few nanomaterial types (Beloin-Saint-Pierre et al., 2018). At the moment it is therefore unclear how LCA could properly address the complexity and dynamism of smart nanomaterials unless more systematic and long-term studies are conducted.

Some authors define ‘sustainable nanotechnology’ as *“green chemistry applied to nanotechnology”* with the aim of maximising functional and economic performance while minimising adverse environmental and human health impacts (Gilbertson et al., 2015). The principles of green chemistry can be applied to produce safer and more sustainable nanomaterials and more efficient and sustainable nano-manufacturing processes. At the same time, nanotechnology is crucial for green innovation and green growth and seen as a mean towards manufacturing more sustainable non-nano materials and products or to help solve economic, environmental and societal issues such as energy shortage or water remediation (Organisation for Economic Co-operation and Development (OECD), 2013; Mauter et al., 2018). However, the energy, waste and resource extraction costs associated with the production of the materials used in green nanotechnology applications remain uncertain and need further research and assessment to assure their responsible development (Organisation for Economic Co-operation and Development (OECD), 2013).

In this context, incorporating life cycle thinking as early in the product development process as possible is key to obtain a complete picture of benefits and negative implications of nanomaterials and nano-enabled products (Dhingra et al., 2010). Less data intensive approaches aimed to inform decisions on material selection already at the design phase have been proposed. For example, Falinski et al. (Falinski et al., 2018) expanded the Ashby material selection charts to include metrics characterising inherent toxicity (zebrafish data), economic performance (price) and environmental life cycle impacts (cumulative energy demand) in addition to function.

For better societal acceptance of smart nanomaterials it is key to know the relationship between nanomaterial properties, their functional performance and possible adverse effects for human health and the environment (Falinski et al., 2018). This is even more crucial for emerging (smart) nanomaterials, whose sustainability is considered to be harder to measure than for traditional nanomaterials due to their increasing complexity and enabling nature (Subramanian et al., 2010). Advanced materials are not necessarily more sustainable than conventional ones. As pointed out by Rycroft et al. (2019), the sustainability score of advanced materials depends on the extent to which the individual components of the score, i.e. economy, environment and society, are valued by an assessor, and which sub criteria contribute most to an improvement in a product's sustainability. This, of course, applies to nanostructured or nanoscale advanced materials, including also smart nanomaterials.

## 4 Are smart nanomaterials safe?

Despite the growing interest towards smart nanomaterials in fields that go beyond biomedical applications, there seems to be little research addressing their potential toxicological impact on human health and the environment, which may originate from their current and future use. Over the last 15 years extensive public and private funding has facilitated progress in understanding the possible risks resulting from passive nanomaterials. The European Commission, for instance, has invested more than 300 million Euro in nanosafety related projects since the beginning of the Fifth Framework Programme (FP5), started in 1999. However, those efforts were not designed to address effects caused by nanomaterials that evolve over time in response to certain stimuli. Accordingly, there are gaps and uncertainties that remain to be addressed. Knowledge and techniques developed for safety assessment of passive nanomaterials may be appropriate for active ones at least to some extent but they need a thorough assessment.

Back in 2007, the International Risk Governance Council (IRGC) raised concern that the higher complexity and dynamic behaviour of active nanostructures compared to passive ones could pose new or increased risks and, at the same time, require a higher level of knowledge and ability to assess those risks (International Risk Governance Council (IRGC), 2007). More recently, some authors have reiterated that the ability of active nanomaterials to change or evolve their state during use may influence their risk assessment and governance (Subramanian et al., 2014; Howard, 2011; Suominen et al., 2016). A survey pointed out issues associated with stability, decomposition and unexpected environmental behaviour together with concerns over consumer exposure when these materials will be scaled up and produced for the market (Sadowski and Guston, 2016).

A detailed analysis aimed at uncovering possible new risks and challenges for the traditional risk assessment paradigm posed by active nanostructures was carried out for three case studies (foldable and self-replicating DNA nanostructures, stimuli-responsive self-assembling proteins, and photo-activated miRNA delivery systems for gene modulation) (Kuzma and Roberts, 2016). The authors concluded that active nanostructures challenge the existing risk assessment frameworks: they behave *“somewhere between a chemical and a living organism”*; they change during their lifetime and may pose different hazards; *“they often contain molecules that can integrate with the hosts' bio-machinery (e.g. DNA fragments and proteins)”*; there is a large variety of possible active nanostructures and, at the same time, a lack of safety data about them.

For nanohybrids, Saleh et al. (2015) concluded that the current risk assessment framework is valid but recommended to implement a comprehensive strategy in order to: 1) identify possible new emergent properties compared to those of the parent materials (e.g. higher dimensionality, greater stiffness); 2) identify those properties that determine fate and toxicity; and 3) evaluate stability and integrity in environmental conditions. In addition, for adaptive nanohybrids, it cannot be neglected that after release these structures encounter a complex environment with coexistence of multiple stimuli and continuous changes in stimuli composition, which will have an unforeseen influence on their environmental and toxicological behaviour (e.g. aggregation/deposition, transformation, uptake and bio-distribution) and introduce additional uncertainty in risk assessment (Plazas-Tuttle et al., 2015). The authors recommend monitoring the dynamic evolution of the coating under changing stimuli in different exposure scenarios, taking into account that equilibrium assumptions need to be replaced with time-dependent variations in surface properties. A shift to predictive modelling, capable of reflecting the heterogenic and dynamic nature of both materials and environmental systems, is envisaged. An effort in this direction has already been done in caLIBRAte (www.nanocalibrate.eu/home) and SmartNanoTox (www.smartnanotox.eu) projects.

## 5 Is Safe-by-Design applicable to smart nanomaterials?

The concept of SbD for nanomaterials and nano-enabled products has emerged in the European Union, along with the development of tools for its implementation in industrial manufacturing processes. Since 2013, the Directorate-General for Research and Innovation (DG RTD) of the European Commission has funded a series of research projects (the ongoing and ended projects overview is available within the Nanosafety Cluster, www.nanosafetycluster.eu) aimed to develop a SIA for nanomaterials, which integrates the two complementary concepts: SbD and RP (Soeteman-Hernandez et al., 2019). SbD was initially formulated for nanomaterials in the flagship project NANoREG and defined as a process enabling an early stage application of the precautionary principle by means of considering health and environmental safety in addition to function in the design phase of a material or product (Gottardo et al., 2017). The concept uses as a backbone the Cooper Stage - Gate model, which divides the innovation process into a predefined set of stages, moving from new product ideas up to introduction into the market and beyond (recycling and re-use). Each stage includes specific activities and the progression from one stage to the next one is regulated by a gate, where the innovation project is judged according to a set of criteria. Decisions at each stage are always based on balancing expected risks, costs and benefits. In Safe-by-Design, six indicators are used from the first stage to assess the potential impact on human health and the environment of a new nanomaterial. Further development of the concept continued in Prosafe and NanoReg2 by promoting the implementation of SbD through the elaboration of a safety dossier and a safety profile at the research and development stage (Kraegeloh et al., 2018) and integrating it with Regulatory Preparedness so as to have a robust but flexible regulatory framework able to keep pace with innovation in nanotechnology (Jantunen et al., 2018b; Soeteman-Hernandez et al., 2019). The terminology behind is now being harmonised at international level in a dedicated OECD project (Organisation for Economic Co-operation and Developemnt OECD, 2020). Furthermore, the Technical Committee 352 (Nanotechnologies) of the European Committee for Standardization (CEN/TC352) has adopted a Preliminary Work entitled “Nanotechnologies - Safe-by-Design concept dedicated for nano scale materials (MNM) and products containing nanomaterials” (CEN/TC352, 2019).

Currently the International Organisation for Standardization (ISO) has not yet developed standards directly aiming at SbD. However, ISO has a liaison with VAMAS (Versailles Project on Advanced Materials and Standards) and publishes Technology Trends Assessments based on the work of VAMAS. Certain currently active Technical Working Areas of VAMAS, such as TWA 33 (Polymer Nanocomposites), TWA 36 (Printed, flexible and stretchable electronics) and TWA 40 (Synthetic Biomaterials), via this liaison may provide pre-standardization work to ISO, related to safe design of advanced materials including smart nanomaterials.

Two projects under the European Union's Horizon 2020 Framework Programme with a total budget of nearly 15 million Euro are foreseen to start before the end of 2020 with emphasis on tools implementing Safe-by-Design for ‘multi-component nanomaterials’, including smart nanomaterials (European Commission, 2020d). Moreover, new activities have recently been proposed under the future European Union's Horizon Europe Framework Programme 2021–2027 aimed to promote research and development on innovative materials that are safe and sustainable by design as well as compliant with the circular economy (European Commission, 2019b, European Commission, 2019c; van der Waals et al., 2019). Importantly, Safe-by-Design is a key tool in the European Union's Circular Economy Action Plan, employed to facilitate *“the progressive substitution of hazardous substances*” (European Commission, 2020a). Also the Chemicals Strategy for Sustainability states that “*Moving to safe and sustainable-by-design chemicals, including to sustainable bio-based chemicals, and investing in finding alternatives to substances of concern is crucial for human health and the environment, as well as an important precondition for reaching a clean circular economy*” (European Parliament, 2020). It is therefore anticipated that future research activities in the European Union will examine whether the current approach to Safe-by-Design covers the dynamic features of smart nanomaterials too and, if not, how to adapt it and provide manufacturers and regulators with the appropriate tools for its implementation.

## 6 Is the regulatory framework ready for smart nanomaterials?

The introduction of a new technology often creates new challenges for legislators, especially when its functionalities, commercially promoted as benefits, raise concerns about human health and environmental risks. In such cases, new legislation or adaptation of the existing one may be necessary. Regulators need to be aware of upcoming new materials, technologies and innovations from the early stages of the innovation process on to understand whether the existing legislation addresses all relevant aspects regarding their human and environmental safety. The RP concept as developed within the SIA aims to improve the foresight of regulators and thus facilitate the development of more adaptable legislation that can keep up with the pace of knowledge generation and innovation in the field of nanotechnology. This concept can in principle be extrapolated to any type of application or material. RP also aims at enhancing the regulatory certainty for innovative products. As an example, in a recent survey industry has expressed hesitation to fully engage in nanotechnology development because of the uncertain regulatory landscape and fear that it could develop into irrational rather than scientifically sound provisions (Sadowski and Guston, 2016). The lack of appropriate risk assessment and regulation to address safety concerns is considered among the possible barriers to the implementation of smart nanomaterials in agriculture, which is a just now emerging technology (Lowry et al., 2019). To overcome this, it is crucial to identify already at the design phase the nanospecific properties that produce beneficial functions and those that may cause adverse effects. In addition, considering the potential impacts throughout the whole life cycle of these new solutions compared to the conventional ones could facilitate their sustainable application (Lowry et al., 2019). In their assessment of whether existing risk frameworks for chemicals and nanomaterials need adaptation or transformation to cover active nanostructures, Kuzma and Roberts (2016) conclude that risk frameworks need to include the use of *“life-cycle thinking, systems thinking, and probabilistic analysis (e.g. engineering concepts, system dynamics models, fault trees, event trees)”* and move from a deterministic to a probabilistic interpretation of risk. They also underline that the concept of risk would need to be broadened to address the social and ethical impacts that the new risks of active nanostructures may pose. Indeed, safety goes hand in hand with sustainability and there is now the necessary political will for a systemic and transformative transition to a circular economy in the EU and possibly beyond. However, this requires also to implement the RP concept, and for smart nanomaterials it means to analyse first the existing regulatory framework for its preparedness and then close possible regulatory gaps by appropriate legal actions.

### 6.1 REACH

ECHA has recently evaluated the applicability of the REACH Regulation to next generation nanomaterials, which are stimuli-responsive materials and structures. They concluded that the identification and characterisation requirements for nanoforms specified in Annex VI of the REACH Regulation do not capture the dynamic dimension of these new applications. As a consequence, further guidance on how to consider the intended function and the changes that occur as a response to external stimuli in a registration dossier would be needed (European Chemicals Agency (ECHA), 2019), and not only for nanomaterials but for other stimuli-responsive materials as well. To this end, new analytical methods may also be necessary for an appropriate characterisation of nanostructures and nanostructured materials. ECHA also raised concern that nanostructures and nanostructured materials may not be covered by the European Commission's recommended definition of the term nanomaterial, which is used for the identification of nanoforms under REACH (European Chemicals Agency (ECHA), 2019). The definition uses the external dimension of constituent particles as the only criterion identifying a nanomaterial. Only solid particles fall under the definition as the dynamic nature of fluid particles does not allow the determination of their external dimensions (Rauscher et al., 2019). As the European Commission recommended definition of nanomaterial does not consider function and dynamism, which are inherent features of smart nanomaterials, there seems to be the need of applying additional criteria that trigger an appropriate safety assessment within the scope of REACH. On the other hand, the European Commission recommended definition of nanomaterial does not prejudge nor reflect the scope of application of any piece of the European Union legislation or of any provisions potentially establishing additional requirements and may be tailored to the needs of a specific legal act. This provides the possibility to introduce changes in the definition of the term nanoform under REACH to cover smart nanomaterials, if deemed necessary.

### 6.2 Novel foods

Nanomaterials are either implicitly or explicitly covered by several pieces of legislation in agriculture, feed and food sectors (Amenta et al., 2015; Rasmussen et al., 2019). The Novel Food Regulation (Plazas-Tuttle et al., 2015) and the Regulation on the Provision of Food Information to Consumers (FIC) (European Commission, 2019c) were revised in 2015 and now include amended provisions for nanomaterials. If a smart nanomaterial is used in food, this considered a novel food and falls within the scope of the Novel Food Regulation. This is because nanomaterials were not used for human consumption to a significant degree within the Union before 15 May 1997, the cut-off date established in the Novel Food Regulation. The FIC Regulation provides explicit provisions for nanomaterials, and the list of ingredients on the food package must include the word ‘nano’ in brackets after the name of the ingredient. Novel foods, and hence smart nanomaterials, must be explicitly authorized to be placed on the European Union's market in food applications. One condition for the latter is that it does not pose a safety risk to human health.

To address this, the European Food Safety Authority (EFSA) published guidance on risk assessment of the application of nanoscience and nanotechnologies in the food and feed chain (Hardy et al., 2018). This guidance applies not only to engineered nanomaterials as defined in the Novel Food Regulation, but also to other materials containing particles that retain properties characteristic of the nanoscale. Such materials need to be assessed for safety and fulfil specific requirements (Hardy et al., 2018). The assessment should consider absorption, distribution, metabolism, excretion, nutritional information, toxicological information and allergenicity and therefore covers the changes of the material when it is in contact and interact with body fluids and tissues. It therefore covers possible changes of a smart nanomaterial resulting from its stimuli-responsive properties.

### 6.3 Food packaging

The European Union legislative framework has a specific Regulation on active and intelligent materials and articles intended to come into contact with food, including packaging (The Commission of the European Communities, 2009). Whereas traditional food packaging is inert by design, active and intelligent food contact materials deliberately interact with food and are able to extend the shelf-life of food products by maintaining or improving the condition of packaged food. This can be done by releasing or absorbing substances to or from the food or its surrounding environment (e.g. an antimicrobial agent), or by indicating the expiry of food through a labelling system that, as an example, changes colour when the maximum shelf life or storage temperature is exceeded (freshness monitoring). The migration of potentially harmful substances from the packaging to food above safety established limits is the main concern associated with food contact materials but the deliberate interaction of active and intelligent packaging materials with food poses new challenges to the evaluation of their safety as compared to the traditional packaging materials (Dainelli, 2008). To this end, the European Food Safety Authority (EFSA) requires that applicants submit in their dossiers information on intended function, manufacturing process and physicochemical properties of the active or intelligent material, the intended use of the active and/or intelligent materials (types of foods and conditions of time and temperature), migration data and toxicological data about the active or intelligent substance, its impurities and degradation products (European Food Safety Authority (EFSA), 2009). Only substances that are included in the Community list of authorized substances may be used in components of active and intelligent materials and articles. Inclusion into the Community List is based on a case-by-case risk assessment and for nanomaterials no exemptions to this exist (Rasmussen et al., 2019). Nanomaterials are also not covered by the functional barrier concept and related exemptions in multilayer food contact materials. In the case that the functional barrier is in contact with the food matrix, specific substance migration requirements have to be met. Thus, nanomaterials can only be used in multilayer food contact materials after a case-by-case risk assessment and explicit authorization (Amenta et al., 2015).

### 6.4 Cosmetics

Cosmetic products are regulated under the Cosmetic Products Regulation 1223/2009, which specifically addresses and defines a nanomaterial as “*an insoluble or biopersistent and intentionally manufactured material with one or more external dimensions, or an internal structure, on the scale from 1 to 100 nm*”. This definition preceded the overarching one recommended by the European Commission in 2011 and was tailored to address specific aspects of cosmetic products. It takes into account not only risk considerations, intentionality of manufacture and solubility of the material but it also defines a nanomaterial not only by the size of the external dimensions of a particle but also by the size of the material internal structure. Thus it may cover to a certain extent stimuli-responsive nanostructured materials designed to be used in cosmetic products (Guan et al., 2019). Before being placed on the European Union's market a cosmetic product has to be notified to the European Commission. A product containing a nanomaterial as an ingredient should be notified to the European Commission six months prior to being placed on the market and in case of concerns over safety, the European Commission will refer it to the Scientific Committee on Consumer Safety (SCCS) for scientific opinion. The Regulation requires applicants to submit information on quantity of nanomaterial in the product, size of particles, physical and chemical properties, intended function, toxicological profile and exposure conditions. The risk assessment of each nanomaterial ingredient needs to be carried out on a case-by-case basis. Furthermore, any nanomaterial ingredient has to be labelled with the suffix ‘nano’ in brackets after the ingredient name. In 2019, the SCCS updated the guidance on safety assessment of nanomaterials in cosmetics, which now explicitly addresses nanocarriers and nanoencapsulated materials (Scientific Committee on Consumer Safety (SCCS), 2019). The assessment of such systems should consider not only the safety of the individual components (e.g. the encapsulating material and the encapsulated contents) but also the safety of the overall entity. The assessment should provide a clear description of the entity in terms of chemical composition, purity, concentration, physicochemical properties, stability, and dermal penetration of both components as well as intended function and uses of the overall entity. Potential toxicological effects and exposure estimates under foreseeable use conditions should be reported for each component and for the nano-encapsulated entity as a whole. These requirements may capture to some extent the dynamic nature of this type of smart nanomaterials. The environmental risk assessment of cosmetic products and ingredients has to be carried out according to REACH provisions. As discussed above, REACH may not fully cover the specific features of smart nanomaterials. Additionally, the definition of the term nanomaterial in both regulations differs and this may lead to an ambiguous situation in which an ingredient that is considered as a nanomaterial under the Cosmetic Products Regulation would not be considered as a nanoform under REACH, thus triggering different requirements. Furthermore, if the nanocapsule or nanocarrier is not an outcome of a reaction process but only the result of a physical mixing, each chemical substance that compose this system has to be registered and assessed separately. This may result in a set of scientifically valid risk assessments which however will not reflect the reality and will not appropriately address the complex and dynamic nature of smart nanomaterials.

All things considered, the European legislation covering food and cosmetic sectors seems to be more prepared than other sectors to address functions and properties of smart materials, structures and devices used in food, food packaging and cosmetic products.

## 7 Discussion and conclusions

Some applications of smart nanomaterials in food, food packaging and cosmetics have already been implemented whereas others could soon reach the market in other sectors such as agriculture. At the moment it is unclear how to measure the sustainability of smart nanomaterials and products enabled by smart nanomaterials. Performing LCA studies as early in the development process as possible is key to obtain a complete picture of their benefits and negative implications but hampered by current gaps in assessing complexity and dynamism of smart nanomaterials. There are also concerns over the safety of these materials as the ability of evolving in response to an external stimulus may pose additional risks to human health and the environment. Scientists and authorities agree that the existing uncertainties and lack of proper methodologies is a challenge for risk assessment and governance of smart nanomaterials. As their safety and sustainability are not well-understood or characterised, current risk assessment and regulatory frameworks are probably not ready to unveil and manage specific risks. A transparent and consistent exchange of information and dialogue among all stakeholders (e.g. developers, safety assessors and regulators) is needed to address safety and sustainability concerns as well as other societal and ethical concerns, thus enabling the full realisation of the benefits that smart nanomaterials can bring to all industrial sectors and society in general. This may be achieved by creating a ‘trusted environment’ in which innovators and regulators can exchange information in a safe and trusted way (Kraegeloh et al., 2018; Soeteman-Hernandez et al., 2019). There are new EU policy initiatives which envisage the establishment of rules and efficient enforcement mechanisms to ensure the access to the data and their re-use across sectors for the benefit of all while applying clear and fair rules (European Commission, 2020e).

A first step towards this was the launch of the European Observatory on Nanomaterials (EUON) by ECHA in 2017 (funded by the European Commission), which is translated into 23 languages in order for the citizens and industrial stakeholders to easily access updated information about the safety and risk assessment of nanomaterials as well as nanomaterial containing products already placed on the EU market.

In addition, incentives leading to an added value for industry could certainly accelerate data and information sharing. These could be regulatory recognition in the form of e.g. grants supporting investment or marketing or a simplified procedure for entering the market. Furthermore, a standardised label is conceivable that explicitly recognises products for which safety and sustainability have been transparently addressed during their development, as suggested earlier by other authors for the SbD approach (Soeteman-Hernandez et al., 2019; Kraegeloh et al., 2018). The Nanotechnology Industries Association (NIA) already organised a series of workshops with regulators, where industry shared their activities towards safer products and how they meet related regulatory requirements (www.nanotechia.org).

**Fig. 2:**
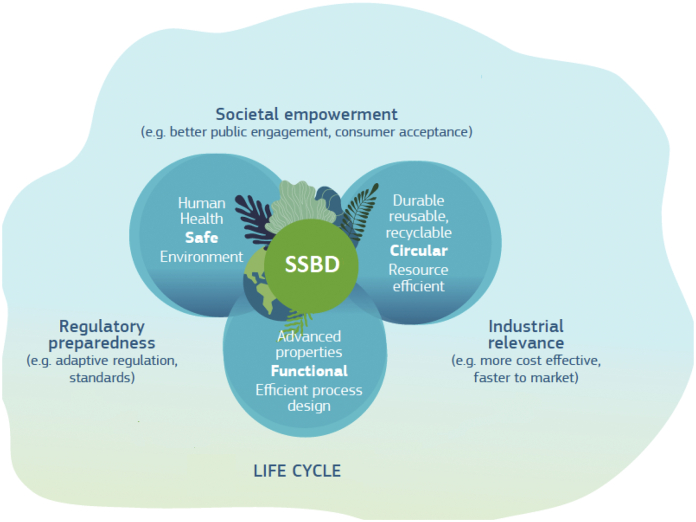
Safe- and Sustainable-by-Design. Materials and products should be safe (for humans and the environment), fit for a climate-neutral, circular and resource efficient economy as well as offer optimal properties and functionality through their life cycle.

Applying the SIA is fundamental but not sufficient to guarantee that new smart nanomaterials are not only safe but also sustainable by design, and therefore compliant with the new political objectives of the European Green Deal, the circular economy and the Chemicals Strategy for Sustainability. Although projects and tools have been developed over the last years (for instance, the online Safe-by-Design platform by TEMAS AG Management Consulting: https://temas.taglab.ch/SbDimplementation/index.php?p=home) and the OECD is carrying out work to internationally harmonise the terminology related to the SIA for nanomaterials, what is missing today is a holistic, systematic and comprehensive approach that allows for considering sustainability aspects hand in hand with safety considerations very early at the material design stage and for balancing safety with impacts on climate, biodiversity and the ability to reuse and recycle resources in a circular economy among others. A leap-change in how environmental and health information at the product and process level is estimated, tracked, communicated, and used is needed to move from the existing scattered landscape of a multitude of concepts addressing isolated aspects of safety and circularity to an overarching framework in which safety and sustainability are the necessary conditions for entry into markets. The ‘Safe- and Sustainable-by-Design’ (SSbD) concept takes a systems approach by integrating safety, circularity and functionality of advanced materials, products and processes throughout their life cycle (Fig. 2).

This concept can be defined as a pre-market approach that focuses on providing a function (or service), while avoiding properties that may be harmful to human health or the environment. As such, it could complement the previously developed SbD approach, a form of prevention-based risk governance, with sustainability considerations, which are fundamental for innovation strategies in line with the key European policy goals (e.g. Green Deal, Action Plan for a Circular Economy, and Chemicals Strategy for Sustainability). Overall, sustainability should be ensured by minimising the environmental footprint of chemicals in particular on climate change, resources use, ecosystems and biodiversity from a life cycle perspective.

As already stated, application of SSbD strategies as early as possible in the industrial research and innovation cycle requires a common, broadly accepted framework defining clear principles. Development of such a framework should follow a stepwise approach:

• Establish the policy framework for the criteria development defining scope, key definitions and links with other EU policies;
• Create an EU-wide SSbD support network aligning actors across the entire value chain (including already existing communities, e.g. consortia working on risk governance of nanotechnology (Gov4Nano: https://www.gov4nano.eu/, RiskGone: https://riskgone.wp.nilu.no/, NanoRigo: https://nanorigo.eu/) and developing modelling frameworks to support the design of safer materials and products (NanoInformaTIX: https://www.nanoinformatix.eu/, NanoSolveIT: https://nanosolveit.eu)) to facilitate knowledge exchange and meeting specific users' needs;
• Identify criteria areas in terms of safety, circularity and functionality (see Fig. 2);
• Define specific criteria for the identified sectors/applications (covering safety, environmental, social and economic aspects).

The framework will necessitate the definition of a research and innovation agenda. Moreover, international standards providing detailed protocols for industry on how to implement SSbD in their specific situations need to be available. In particular, the further development of new and updating of already existing nanospecific OECD test guidelines (TG) and guidance documents (GD) has to be encouraged and supported on industrial level. The European Commission together with 18 European countries are already supporting this development via the Malta Initiative (https://www.bmu.de/en/topics/health-chemical-safety-nanotechnology/nanotechnology/the-malta-initiative/) and also via the EU projects NanoHarmony (https://nanoharmony.eu/) and Nanomet (http://www.oecd.org/chemicalsafety/nanomet/). The availability of harmonised protocols would increase the use of SSbD in industrial settings, including in small and medium enterprises. At the same time, the existing regulatory framework could be revised to explicitly recommend or require the use of SSbD in the development and production of nanomaterials and nano-enabled products, particularly smart ones, in order to effectively address smart nanomaterials' specificities in terms of safety and sustainability. To this end, an anticipatory activity could be initiated to analyse different regulatory options and scenarios to promote the use of SSbD in the European Union (and beyond) while evaluating the impact of each alternative, from the most stringent option of introducing legally binding requirements in existing (or new) regulations to softer approaches such as producing guidance documents or recommendations for industry.

## Author contributions

Stefania Gottardo conceived the study and designed the structure of the paper. She collected the relevant literature, wrote the introduction and the sections on terminology, sustainability and safety of smart nanomaterials. Agnieszka Mech contributed to the design of the paper, she wrote the sections on applications of smart nanomaterials in food, food packaging and cosmetics and included the concept of regulatory preparedness and safe-by-design. She also analysed the applicability of the Cosmetic Products Regulation and the European Commission's definition of nanomaterial to smart nanomaterials. Hubert Rauscher analysed the applicability of the current regulatory framework in the European Union to smart nanomaterials and enabled products and wrote the corresponding section. Juan Riego Sintes supervised and provided extensive feedback. Jana Drbohlavová, Søren Bøwadt and Alexandra Małyska provided applications of smart nanomaterials and considerations on the policy related to sustainability. All authors contributed to the discussion and conclusions section and to the final manuscript.

## Declaration of Competing Interest

The authors declare no competing interests.
